# Impact and mitigation of angular uncertainties in Bragg coherent x-ray diffraction imaging

**DOI:** 10.1038/s41598-019-42797-4

**Published:** 2019-04-23

**Authors:** I. Calvo-Almazán, M. Allain, S. Maddali, V. Chamard, S. O. Hruszkewycz

**Affiliations:** 1 Materials Science Division, Argonne National Laboratory, Argonne, Illinois 60439 USA; 2 Aix Marseille Univ, CNRS, Centrale Marseille, Institut Fresnel, F-13013 Marseille, France

**Keywords:** Imaging techniques, Characterization and analytical techniques

## Abstract

Bragg coherent diffraction imaging (BCDI) is a powerful technique to explore the local strain state and morphology of microscale crystals. The method can potentially reach nanometer-scale spatial resolution thanks to the advances in synchrotron design that dramatically increase coherent flux. However, there are experimental bottlenecks that may limit the image reconstruction quality from future high signal-to-noise ratio measurements. In this work we show that angular uncertainty of the sample orientation with respect to a fixed incoming beam is one example of such a factor, and we present a method to mitigate the resulting artifacts. On the basis of an alternative formulation of the forward problem, we design a phase retrieval algorithm which enables the simultaneous reconstruction of the object and determination of the exact angular position corresponding to each diffraction pattern in the data set. We have tested the algorithm performance on simulated data for different degrees of angular uncertainty and signal-to-noise ratio.

## Introduction

Bragg coherent diffraction imaging (BCDI) is a lens-less technique which explores the local morphology and structural imperfections of micro-crystalline samples, shedding light on topics which are central to material science, for example materials synthesis or device performance^1–7^. It is based on the oversampling of the intensity of the three dimensional (3D) Fourier components surrounding a crystal Bragg peak, which is inverted via phase retrieval. At a Bragg peak, such a distribution of intensity encodes structural information about the crystal in the far-field, including strain fields and dislocations^1,2,8^. BCDI measurements require x-ray beams with a degree of coherence only available at synchrotron sources and free-electron lasers (XFEL). The recent development of a new synchrotron design that promises orders-of-magnitude improvements in coherent flux will enable BCDI measurements with nanometric spatial resolution, opening the door to new areas of exploration in materials science via in-situ experiments in realistic environments and with highly penetrating x-rays (~10–50 keV)^9,10^. However, in envisioning such experiments we can foresee factors that limit this potential, and that are not apparent at the low signal-to-noise ratios (SNR) of current measurements.

In this paper, we focus on the detrimental effect of the angular positioning error of the microcrystalline sample with respect to the incident beam direction at levels commensurate with goniometer uncertainties and slow rotational drift of the sample. Such angular uncertainty can come about when a stationary incident beam illuminates a sample undergoing uncontrolled rotation (as in high-temperature experiments) or imprecise experimental stages, difficult-to-stabilize sample environments or, even, to the torque exerted by the x-ray beam on the sample (the radiation pressure). Examples of this kind of issues are encountered in recent studies of strain distribution in semiconducting nanowires^11^ where the authors attribute some of the spatial features in the reconstructed object to angular uncertainties of the 10% of the nominal angular spacing. The latter determines the reciprocal space interval separating adjacent diffraction patterns in the reciprocal space maps. The authors of ref.^11^ estimate a random RMS of 0.0078 degrees on an angular spacing of 0.02 degrees. BCDI measurements on silicon carbide (SiC) nanoparticles at high temperature reported in ref.^12^ were challenging because of the uncontrolled angular drifts produced by the experimental stage at 900 C. Angular shifts of up to 0.02 degrees were observed over the course of minutes. The uncontrolled rotation of Pd nanocubes due to the radiation pressure is described in ref.^13^.

Current BCDI inversion algorithms are based on the formulation of the *forward problem* which uses the 3D Fourier transformation to retrieve an image of the crystal $\rho$ from the 3D diffracted wave-front ${\rm{\Psi }}$ in the far-field: ${\rm{\Psi }}={ {\mathcal F} }_{3D}[\rho ]$^1,2,14^. Experimentally, the 3D reciprocal space is sampled by a two-dimensional (2D) area detector which measures slices of the 3D diffracted intensity around a Bragg reflection at intersections determined by the angle between the crystal and the incoming beam, *θ*^2^. For current inversion algorithms to produce a high quality image, ${\rm{\Psi }}$ needs to be evenly sampled in each of the three dimensions. While the uniform pixelation of the area detector ensures regular sampling in the detector plane, sampling regularity in the angular orientation of the crystal can be perturbed by mechanical instabilities, as mentioned above. This work is a proof-of-concept demonstrating that these perturbations in angular sampling can be mitigated at high SNR conditions by designing phase retrieval strategies which relax the angular sampling regularity constraint. This approach allows the joint estimation of the object and the set of incident sample angles actually queried in the measurement. We have simulated experimental data with noise levels that can be anticipated in future synchrotron facilities and with various levels of realistic angular perturbation (*δθ*%). To invert this series of data, we propose a *hybrid* strategy which combines standard inversion algorithms with algorithms based on an alternative formulation of the forward problem^4,5,11^ which allows for irregular sampling along *θ*. While our method efficiently determines the position at which the area detector records the diffraction patterns, it does not address directly the issue of continuous angular drifting of the sample during the recording of single diffraction patterns. It rather approximates it by an instantaneous and unknown rotation of the sample, which remains stable during the exposure time. Furthermore, it assumes that the angular uncertainty only occurs in the direction of the rocking curve, and not along other degrees of freedom of the goniometer. This simplification aims to highlight the impact of angular uncertainties in the object reconstruction, without obscuring it by other factors related to a complicated experimental set-up. Our simulations also assume that the beam is fully coherent in the transverse and longitudinal directions. As a test sample we have designed a 200-nm-diameter strained crystal as in a typical BCDI experiment^1,2,15^. We anticipate that the size of the sample should not affect the feasibility of angular sampling corrections, at least for the range of angular uncertainties addressed in this work. A larger crystal requires an adaptation of the sampling conditions to meet the Nyquist criterion^16^. In particular it requires a smaller angular step, and therefore, there is an increase in the angular uncertainty relative to the angular step, making the correction of the angular positioning more challenging. However, a benefit of a larger sample is that the SNR increases improving the efficiency of our approach.

**Figure 1:**
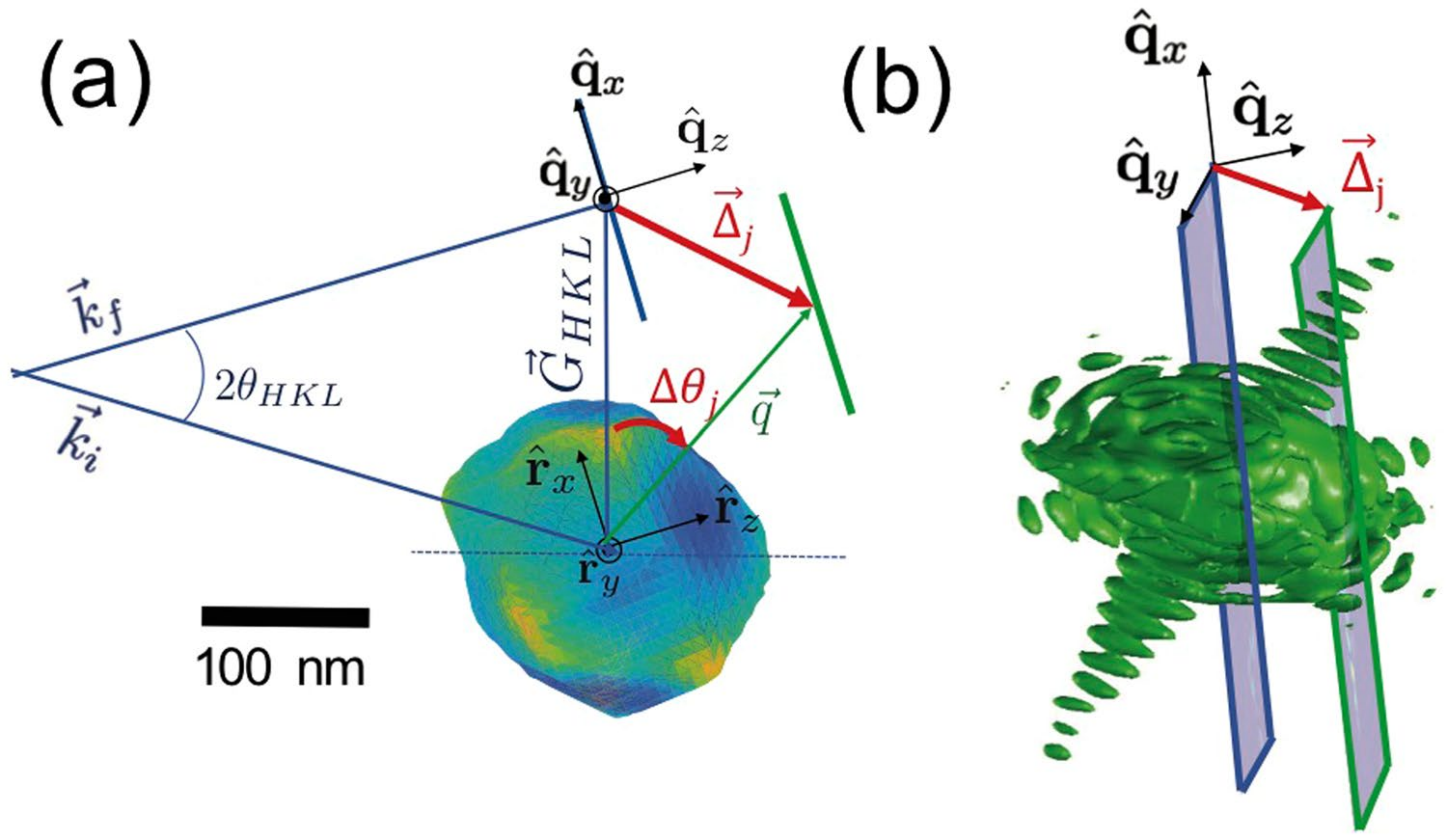
Panel (a) A strained nanocrystal is shown illuminated by a coherent x-ray beam in a symmetric Bragg scattering geometry denoted by the vector ${\overrightarrow{G}}_{HKL}$. The color scale of the nanocrystal surface corresponds to the heterogeneous phase field $\varphi (\overrightarrow{r})$ which encodes the displacement field $\overrightarrow{u}(\overrightarrow{r})$ in the direction of ${\overrightarrow{G}}_{HKL}$ according to $\varphi ={\overrightarrow{G}}_{HKL}\cdot \overrightarrow{u}$^1–3^. The effect of the rocking of the sample by an angle Δ*θ*_*j*_ is displayed in panel (b). The $\hat{\theta }$ direction corresponds to the direction of the vector ${\overrightarrow{{\rm{\Delta }}}}_{j}$ which links the position of the two slices.

The geometry of a simple, but nonetheless realistic strain-sensitive BCDI measurement is described in panel (a) of Fig. 1. A coherent x-ray beam of incident and exit wavectors **k**_*i*_ and **k**_*f*_  (yielding a momentum transfer ${\bf{q}}={{\bf{k}}}_{f}-{{\bf{k}}}_{i}$) is scattered by the 200-nm-diameter strained crystal represented as a 3D isosurface. A Bragg condition is met when the momentum transfer vector **q** coincides with one of the reciprocal space lattice points **G**_*HKL*_ of the crystal, corresponding to the HKL Miller indices of the diffracting planes^8^. The rocking of the sample angle by Δ*θ*_*j*_ about the Bragg angle (*θ*_*HKL*_) displaces the detector measurement plane in reciprocal space by a vector ${\overrightarrow{{\rm{\Delta }}}}_{j}\equiv {{\bf{q}}}_{j}-{{\bf{G}}}_{HKL}$^4,8^. Because the magnitudes of both **q** and **G**_*HKL*_ far exceed that of ${\overrightarrow{{\rm{\Delta }}}}_{j}$ (Fig. 1 is not to scale in this respect), a small angular rocking curve consisting of ~100 even intervals over ~±0.5° results in a series of effectively parallel 2D slices from which a 3D reciprocal space map of the Bragg peak and its surrounding fringes can be resolved so as to satisfy the Nyquist sampling criterion^16^. This is illustrated by way of numerical simulations in panel (b) of Fig. 1, wherein the green intensity isosurface represents a Bragg peak decorated with the fringes arising from the finite size microcrystal featured in panel (a) and the gray planes depict the detector slices at two different rocking angles. The data were synthesized by scanning the crystal in an angular range of ${\rm{\Delta }}\theta =\pm \,0.5^\circ$ in 128 even angular steps. The detector was simulated to be a two dimensional array of 128 × 128 pixels with 55 *μ*m edge size. It is worth clarifying that the ±0.5° angular range scanned by the sample is different from the angular range subtended by the detector, (as in typical BCDI experiments)^1,6^. Following previous experimental approaches^1,6,7^, we assume that the x-ray beam is a plane wave with an energy of 10.4 keV (*λ* = 1.19 Å), a detector to sample distance of 0.5 meters, and a Bragg condition with 2*θ*_*HKL*_ = 73.3 deg, which corresponds to a realistic scattering angles from semiconductor crystals^11^. The diffracted intensity distribution at these two slices is calculated through the alternative formulation of the *forward model* which describes explicitly the wave-field at each slice, ${{\rm{\Psi }}}_{j}$. To obtain a given slice, this model uses a 2D Fourier transformation (${ {\mathcal F} }_{2D}$) that acts on a 3D→2D projection (*R*_*z*_) of the 3D object ($\rho$) modified by a multiplicative phase term, ${\mathscr{Q}}=\exp (i{\overrightarrow{{\rm{\Delta }}}}_{j}\cdot {\bf{r}})$^4,5,11^:

1 $${{\rm{\Psi }}}_{j}\equiv {\rm{\Psi }}(\overrightarrow{q},{\overrightarrow{{\rm{\Delta }}}}_{j},\rho )={ {\mathcal F} }_{2D}\{{R}_{z}[{\mathscr{Q}}({\overrightarrow{{\rm{\Delta }}}}_{j})\rho ]\}$$

In this expression, the key feature is the term ${\mathscr{Q}}$ which enables the calculation of slices of ${\rm{\Psi }}$ corresponding to a set of *arbitrary* incident angles that need not be regular. This is due to the fact that any given sample rotation away from the Bragg angle (Δ*θ*_*j*_) can be expressed as a corresponding displacement of the detector measurement plane by a vector ${\overrightarrow{{\rm{\Delta }}}}_{j}$ in reciprocal space away from the Bragg peak.

**Figure 2:**
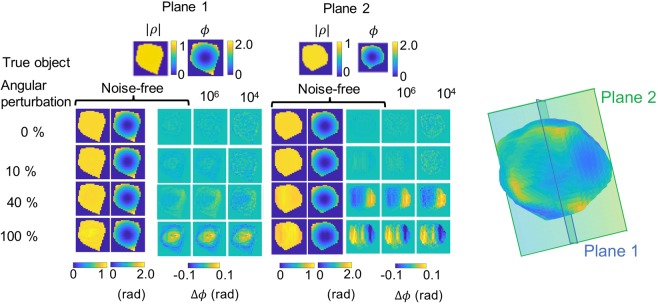
Effect of an increasingly irregular angular grid on the amplitude and the phase on the 3D Fourier inverted image of a data set generated with Eq. 1. Two perpendicular sections of the crystal, represented by the two perpendicular planes in the 3D isosurface, have been displayed to emphasize the volume distribution of the phase artifacts. First and second column: amplitude |$\rho$| and phase *ϕ* of the inverted noise-free data. Third - fourth columns: difference of phase Δ*ϕ* with respect to the original object for the inversion of noise-free data and two SNRs: 10^6^ and 10^4^.

When regular angular increments are presumed but not in fact realized, traditional BCDI phase retrieval methods introduce artifacts in the 3D images of the object. Figure 2 shows the inverse Fourier transformation (which presumes angular regularity) of a data set collection with varying degrees of noise and angular perturbation levels. The diffraction patterns were generated using Eq. 1 applied to the 3D strained nanocrystal featured in Fig. 1. The slices of diffracted intensity corresponded to a set of regularly-spaced angles with added uniformly-distributed angular uncertainties ranging from *δθ* = 10% to 100% of the prescribed regular angular increment of 0.0078°. We introduced Poison-distributed noise and calculated the signal-to-noise ratio (SNR) as the ratio of the power of the diffracted intensity to the power of the noise. Thereby we generated data sets with SNRs ranging from 10^4^–10^6^ which mimic signal levels that are typical of BCDI measurements made at today’s third generation synchrotron facilities (10^4^) up to those anticipated at fourth generation diffraction limited storage ring light sources (10^6^). Columns 3–5 in Fig. 2 show Δ*ϕ*, the phase difference between the Fourier-transform-inverted and the true object. To characterize the magnitude of the resulting phase artifacts, we have calculated the histogram of Δ*ϕ* values within each 3D reconstructed object as a function of each *δθ*% and SNR. The standard deviation *σ of the histogram* is shown in Table 1. In general terms, we find that the introduction of noise systematically broadens the Δ*ϕ* distribution, as expected. The finite value of *σ* in the no-angular noise-free case arises from the numerical evaluation of the analytic expression in Eq. 1 which introduces rounding errors that are propagated in the inverse Fourier transform operation. We also observe that at the higher SNR level, where noise does not corrupt the signal as strongly, phase artifacts arise (*i*.*e*. *σ* broadens) at *δθ* as low as 10%. By contrast, the effect of such a small magnitude of angular perturbation is not nearly as apparent at more moderate SNR characteristic of experiments feasible today. We emphasize that this test does not involve the use of any reconstruction algorithm. The purpose is to show that in *the best case*, when the phases of the object are known, the use of a 3D inverse Fourier transformation which neglects angular uncertainty produces artifacts in the reciprocal space image. Furthermore, these artifacts are pronounced at SNR levels anticipated at fourth generation synchrotron sources which will provide a hundred time increase in coherent photon flux. Thus for a fixed counting time, the spatial resolution and the SNR will be significantly improved. In this regime, the mitigation of angular position uncertainty becomes relevant in the interpretation of results, since heterogeneous phase fields within the image reconstruction are interpreted in terms of internal crystalline strain, defects or dislocations^1–3^. Thus, it is timely to design a strategy for phase retrieval algorithms which can mitigate this problem.

**Table 1:** Standard deviation *σ* in radians of the difference of phase Δ*ϕ* distribution from the 3D inverted object as a function of the percentage of angular uncertainty *δθ* and the SNR.

*δθ*%	*σ* × 10^−3^		
	Noise-free	SNR = 10^6^	SNR = 10^4^
0	3.6	5.9	13.4
10	5.3	7.1	13.8
40	34.3	34.9	36.6
100	44.4	44.7	46.1

**Figure 3:**
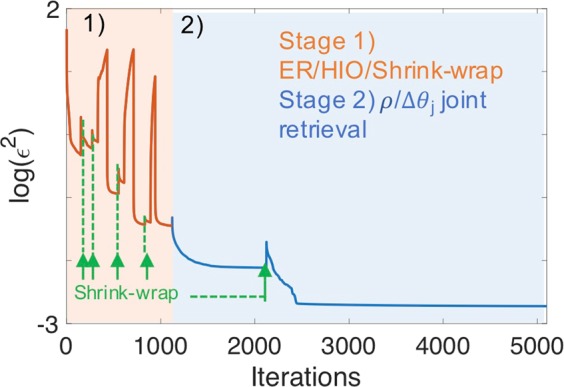
Evolution of the error metric over the stages 1 and 2. The stage 1 (orange area) uses standard ER/HIO/Shrink-wrap with no angle correction to provide a good initial guess. The stage 2 (blue area) refines the object and jointly estimates the angular positions.

In this work, we propose a two-stage *hybrid* strategy to correct for such artifacts which consists of performing a joint retrieval algorithm of $\rho$ and Δ*θ*_*j*_ starting from results yielded by well-known phase retrieval strategies such as error reduction (ER)^17^, hybrid-input-output (HIO)^17^, and shrink-wrap (SW)^18^. The joint retrieval approach in the second stage exploits the flexibility of the forward model in Eq. 1 to treat the set of incident beam angles Δ*θ*_*j*_ as free parameters. In the typical BCDI approach, the error metric ${\varepsilon }^{2}={\sum }_{j}\,|||{{\rm{\Psi }}}_{j}|-\sqrt{{I}_{j}}|{|}^{2}$ can be used to derive a gradient $\frac{\partial {\varepsilon }^{2}}{\partial \rho }$ to guide ER or HIO in the estimation of $\rho$^14,17,19^. In addition, Eq. 1 also allows $\frac{\partial {\varepsilon }^{2}}{\partial {\rm{\Delta }}{\theta }_{j}}$ to be calculated. We can therefore introduce a form of the error metric gradient: $\nabla {\varepsilon }^{2}=\{\frac{\partial {\varepsilon }^{2}}{\partial \rho },\frac{\partial {\varepsilon }^{2}}{\partial {\rm{\Delta }}{\theta }_{j}}\}$ which enables the joint estimation of $\rho$ and the set of Δ*θ*_*j*_ *via* scaled conjugate-gradient (CG) with adaptive steps-size^20^. The simultaneous retrieval of the object and the correction angles is inspired by the position correction method implemented in ptychography^19^. At the start, the object $\rho$ is initialized with uniform random complex numbers, the support is a box half the size of the numerical window, and the set of intensity patterns corresponding to ${{\rm{\Psi }}}_{j}$ are assembled into a 3D array. The first stage consists in a total of 1200 iterations of the ER/HIO phase retrieval algorithms where we periodically update the support size *via* the SW method. Since we observe a variability in the quality of the final reconstruction, we adopted a prescription used in references^7,21^ to design *guided* retrieval algorithms. We repeated the ER/HIO/SW cycle 10 times and we choose the reconstructed object and the support yielding the lowest error metric as the final reconstruction. This reconstruction is the starting point of stage 2, consisting of 4000 iterations with joint estimation of $\rho$ and Δ*θ*_*j*_. The following prescription was used: within a single stage 2 iteration, we first update $\rho$ and then, the array of Δ*θ*_*j*_ values. We performed a last SW of the support at the 2000th iteration, which raised istantaneously the value of the error metric (by removing degrees of freedom), but which allows the further minimization of the error metric. Finally, in order to compensate for the disparity of photon count rates in the detector at each slice, we normalize the gradient for every Δ*θ*_*j*_ by the mean count rate in each diffraction pattern. This normalization enables a more uniform correction at all angles in the rocking curve. In particular, it allows for efficient correction of the angular uncertainty of the high-*q* tails of the rocking curve. Figure 3 summarizes the core concept of such a hybrid approach applied to noise-free data with *δθ* = 10%, showing the two stages of phase retrieval denoted by orange and blue colored regions of the plot.

**Figure 4:**
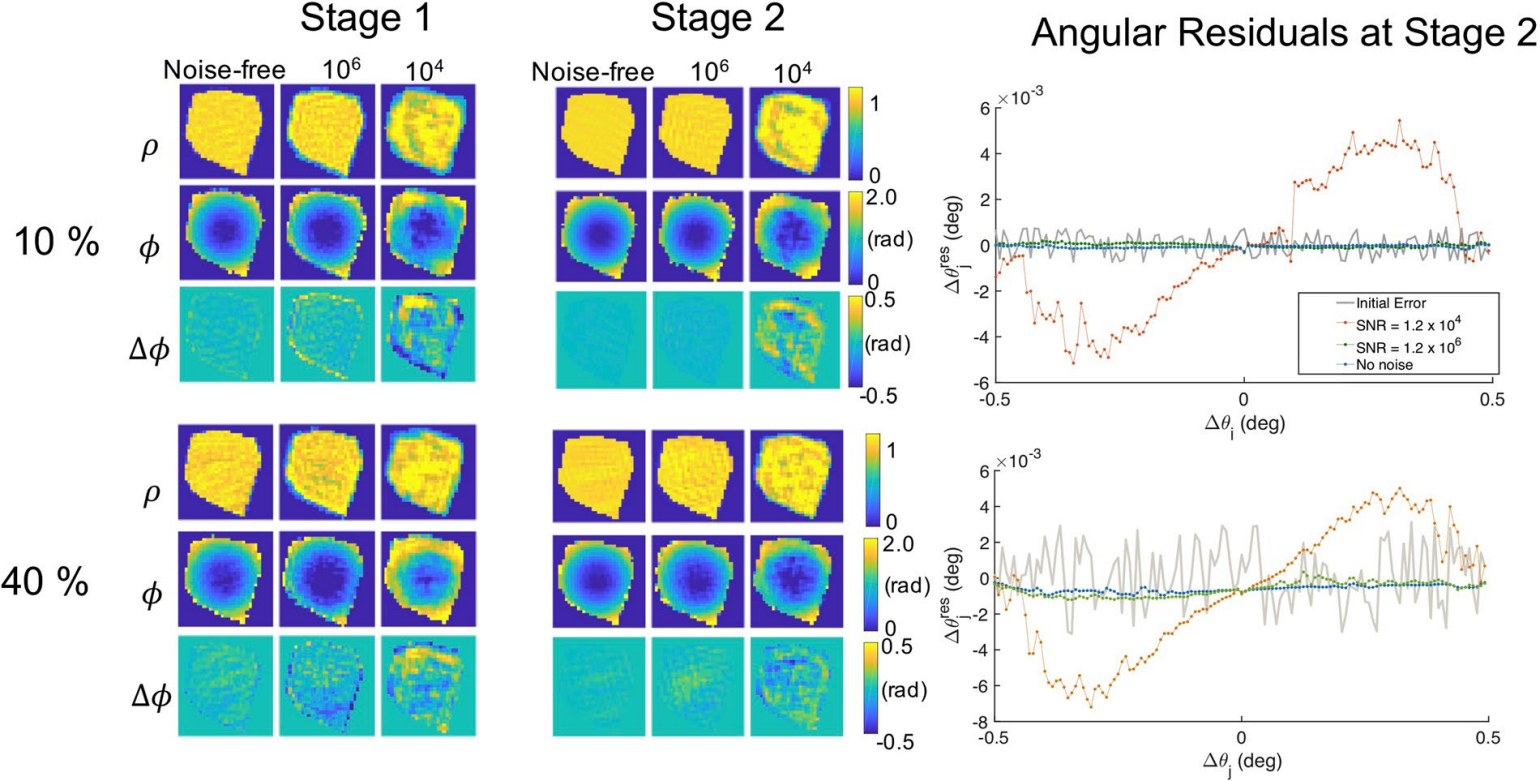
Left panel: Reconstructed objects (amplitude |$\rho$|, phase *ϕ* and difference of phase Δ*ϕ* at the slice of the 3D volume corresponding to plane 1 in Fig. 2) from data affected by 10% and 40% angular uncertainties and different degrees of noise. The retrieved object in stage 1 is shown after 1200 iterations of ER/HR/SW and the object in stage 2 is the result of 4000 iterations of the hybrid strategy. Right panel: Angular error ${\rm{\Delta }}{\theta }_{j}^{res}$ at the initial and final iteration of stage 2 obtained from the different SNRs data disturbed with a 10% and a 40% of angular uncertainty.

In order to assess the performance and limits of our reconstruction method, the two-stage phase retrieval strategy was tested under the above SNR conditions for different levels of angular uncertainty, up to 100% of the nominal angular increment of 0.0078°. The resulting *σ* values at the end of stage 1 and 2 for all cases considered in this work are summarized in Table 2. The results after stage 1 highlight that there are two factors which are detrimental for the reconstruction quality: noise and angular uncertainty. In the case of low SNR, the dominant factor is the noise. In contrast, in the high SNR regime, the angular uncertainty becomes prevalent. In general terms, we find that *σ* decreases significantly after stage 2 in the high SNR case, while at low SNR and highest *δθ* case there is not a qualitative improvement between the reconstruction after stages 1 and 2. Thus, the hybrid optimization strategy is most effective for mild perturbations upon a regular grid (up to 100%) and high SNR. It is in this regime that we expect to improve the quality of the reconstruction. The high SNR regime can be obtained in today’s synchrotrons as illustrated in ref.^22^ for a crystalline sample with a similar size that the one chosen in this work. We note that a few orders of magnitude can be gained or lost with the use of different focusing optics, exposure time, detector efficiency or crystalline material. Finally, the advent of 4th generation synchrotron sources, delivering a gain of 2 orders of magnitude in the coherent flux^9^ justifies the SNR values of the simulations. An example of the reconstructed object for data with *δθ* = 10% and 40% can be found in Fig. 4. For the 10% angular perturbation and a SNR of 10^6^, the reduction of *σ* from 0.14 to 0.03 radians corresponds to a reduction in the spurious displacement from 0.05 to 0.008 Å, value which is negligible in comparison to measured displacement fields^2^. A similar value is found for *δθ* = 40% and high SNR, demonstrating the feasibility of mitigating angular artifacts due to relatively high angular uncertainties in high statistical quality data. Furthermore we do not observe any cross-talk between the phase of the object and the angle value. Therefore the hybrid strategy is satisfactory in removing angular artifacts and not actual structural features.

**Table 2:** Standard deviation *σ* in radians of the difference of phase Δ*ϕ* distribution of the reconstructed 3D object as a function of the percentage of angular uncertainty *δθ* and the SNR. Stages 1 and 2 correspond to the results delivered by the ER/HIO/SW and the joint phase retrieval algorithms respectively.

*δθ*%	Noise-free		SNR = 10^6^		SNR = 10^4^	
	Stage 1	Stage 2	Stage 1	Stage 2	Stage 1	Stage 2
10	0.06	0.01	0.14	0.03	0.26	0.15
40	0.09	0.04	0.17	0.07	0.27	0.13
100	0.19	0.18	1.91	0.56	1.59	1.68

We observe that for SNR of 10^4^, the presence of noise affects the correct estimation of the support in stage 1, altering the quality of the final reconstruction in stage 2. It also prevents the effective determination of angles. This can be seen in the right panel of Fig. 4 which shows for the same *δθ*s disturbed data, the evolution of the angular residuals ${\rm{\Delta }}{\theta }_{j}^{res}={\rm{\Delta }}{\theta }_{j}^{true}-{\rm{\Delta }}{\theta }_{j}$ over the course of stage 2. For lower SNR (10^4^) and both angular perturbations, the angular residuals adopt a sinusoidal function of the nominal angle Δ*θ*_*j*_. In the vicinity of ${\rm{\Delta }}{\theta }_{j}=0$, the function can be approximated by a line of slope of 9.8 × 10^−3^. A finite slope in the angular residuals indicates a differently spaced regular sampling grid in reciprocal space. This variation of the pixel size in reciprocal space changes the total size of the object in the conjugated direction of real space^23^. Thus, this behavior of the angular residuals manifests itself as a scaling of the object of 1% in the conjugate direction of ${\overrightarrow{{\rm{\Delta }}}}_{j}$. Conversely, with increasing SNR, the fixed observation angle and pixelation of the detector suppresses this scaling artifact in the object. As a result, in our tests, the incident angle residuals tend towards zero for SNR of 10^6^ and noise-free data.

The conclusion is that BCDI reconstructions from high SNR (>10^5^) data sets and mild angular perturbation ($\leqslant$40%) are expected to show significant improvement when employing joint estimation of $\rho$ and Δ*θ*_*j*_. In the case of low SNR data and large angular perturbations ~100%, we should invoke a more sophisticated joint estimation algorithm which incorporates a global optimization approach such as simulated annealing^24^. Finally, we note that the simulated beam was a plane wave, and therefore we have not addressed the question of the joint estimation of object and angles with a curved wavefront. In principle, the wavefront curvature acts as a global phase profile of the object and could be handled with an adapted algorithm. We also emphasize that we have approximated the continuous angular drift of the sample as an instantaneous rotation of unknown angle (that the algorithm is able to retrieve). Our approach paves the way to design more advanced algorithms which mitigate simultaneously a variety of detrimental factors (not taken into account in the present work) such as the partial coherency of the beam^22^, the effect of the oversampling ratio and the detection dynamic range^23^, the binning of the signal within the area of a finite size pixel^25^, or the continuous drift of the sample during the measurement^26^.

Overall, the set of numerical tests presented here demonstrates the feasibility of post-hoc determination of uncertain angular sampling and irregularity in BCDI rocking curve measurements. Through the design of a two stage *hybrid* phase retrieval approach we are able to perform the joint estimation of a strained crystal $\rho$ and the actual angular sampling grid Δ*θ*_*j*_. This approach presumed no prior knowledge of the object or the support, and assumed regular angular sampling of the diffracted intensity as a starting point. The test of our approach for uniformly distributed angular uncertainties ranging between 10% and 100% and two SNRs, 10^4^ and 10^6^, shows that the method is capable of retrieving the object and the set of incident angles for mild angular perturbations (≤40%) without cross-talk with the object phase. Furthermore, we obtain a good recovery of the incident angles from data with SNRs above 10^6^, but we observe an artifact in the Δ*θ*_*j*_ estimation for SNRs ~ 10^4^. Finally, we note that we have designed a BCDI experiment on a 200 nm strained nanocrystal with a simplified but realistic scattering geometry. The purpose is to underline the basic concept of the impact of angular positioning errors in BCDI reconstructions. In order to apply our approach to experimental data, a full description of a six axis goniometer is required. Such a description involves a non-trivial generalization of the scattering geometry in three dimensions. Furthermore, it also depends on the synchrotron beamline where the experiment is performed and, therefore, needs to be customized for every particular experiment.

The ability to relax the requirement of regular angular sampling in BCDI experiments and to correct for angular uncertainties can open the door to new realms of exploration. Such studies will be relevant to crystalline materials under conditions that induce sample instabilities such as high temperature or high pressure. This work lays the groundwork for such an approach, and is especially timely because the latest generation of high-brightness diffraction-limited storage ring synchrotron sources are now coming online worldwide. Furthermore, our approach can be easily incorporated in Bragg ptychography algorithms, enabling the simultaneous retrieval of the object and the correction of uncertainties in the beam positions and the angle. Another direction of improvement is the combination of angular uncertainties and partial coherence of the beam corrections. All these advances in reconstruction strategies and phase retrieval algorithms further broaden the envelope of possible high-resolution in-situ imaging experiments of crystalline matter.

## Data Availability

The data reported in this paper are available upon request. All code, including the reconstruction algorithm, is also available upon request.
